# Does an Oblique/Slanted Perspective during Virtual Navigation Engage Both Egocentric and Allocentric Brain Strategies?

**DOI:** 10.1371/journal.pone.0049537

**Published:** 2012-11-28

**Authors:** Julien Barra, Laetitia Laou, Jean-Baptiste Poline, Denis Lebihan, Alain Berthoz

**Affiliations:** 1 Laboratoire Mémoire & Cognition, Institut de Psychologie, Université Paris Descartes, Boulogne-Billancourt, France; 2 Laboratoire de Physiologie de la Perception et de l'Action, CNRS UMR 7152, Collège de France, Paris, France; 3 NeuroSpin, I2BM, CEA, Gif-sur-Yvette, France; George Mason University/Krasnow Institute for Advanced Study, United States of America

## Abstract

Perspective (route or survey) during the encoding of spatial information can influence recall and navigation performance. In our experiment we investigated a third type of perspective, which is a slanted view. This slanted perspective is a compromise between route and survey perspectives, offering both information about landmarks as in route perspective and geometric information as in survey perspective. We hypothesized that the use of slanted perspective would allow the brain to use either egocentric or allocentric strategies during storage and recall. Twenty-six subjects were scanned (3-Tesla fMRI) during the encoding of a path (40-s navigation movie within a virtual city). They were given the task of encoding a segment of travel in the virtual city and of subsequent shortcut-finding for each perspective: route, slanted and survey. The analysis of the behavioral data revealed that perspective influenced response accuracy, with significantly more correct responses for slanted and survey perspectives than for route perspective. Comparisons of brain activation with route, slanted, and survey perspectives suggested that slanted and survey perspectives share common brain activity in the left lingual and fusiform gyri and lead to very similar behavioral performance. Slanted perspective was also associated with similar activation to route perspective during encoding in the right middle occipital gyrus. Furthermore, slanted perspective induced intermediate patterns of activation (in between route and survey) in some brain areas, such as the right lingual and fusiform gyri. Our results suggest that the slanted perspective may be considered as a hybrid perspective. This result offers the first empirical support for the choice to present the slanted perspective in many navigational aids.

## Introduction

When we arrive in an unknown city, we can learn to navigate either by storing information on the sequence of streets that we take and the corresponding landmarks or episodes (“egocentric” or “kinaesthetic” route strategy), or we can obtain a map of the city and plan and store our travel on a map-like “survey” view of the city (“allocentric” strategy) [1], [2]. Egocentric route-based knowledge is defined as knowledge of spatial layout from the perspective of a ground-level observer navigating the environment and storing sequences of combinations of views and landmarks. By contrast, survey (allocentric) knowledge is characterized by an external perspective, such as an aerial or map-like view, allowing direct access to the global spatial layout. These two strategies develop during childhood [3], [4]. We can also combine these two strategies, which improves our ability to find new routes and shortcuts, and many recent studies have been devoted to their neural basis [5]–[9]. Most studies in this domain have been performed using virtual reality, but new experiments using real locomotion have also shown that it is possible to dissociate these strategies using behavioural paradigms [10].

Both perspectives provide information about spatial layout, but they can have different behavioural consequences, since map learning is superior for judgments of relative location and straight-line distances between objects, and route perspective learning is superior for estimating route distances [11], [12]. Furthermore, the two perspectives involve different brain areas. Right hippocampus activation has been found for allocentric imagery, left hippocampus activation for route learning, and bilateral activation of parahippocampal gyrus for route imagery [13]. Mellet et al. [14] were the first to compare brain activation during mental imagery after route and survey learning. However, they used the visualisation of previously learned environments and not the encoding of novel ones. Studies in humans [5]–[9] have since identified a network of brain areas for processing spatial information from different perspectives, including parahippocampal cortex, hippocampus, posterior cingulate, precuneus, retrosplenial cortex, and premotor cortex (see [15], [16] for a review). In addition, different brain areas are involved in viewer-centred, object- centred and landmark-centred judgments about object location in environmental space [17].

The complexity of the network used for spatial processing is also illustrated by recent results concerning lateralisation and gender differences. For example, it has been suggested that there is a lateralisation of function: according to this view, in humans the left hippocampus is mainly involved in sequential and episodic (route) coding of paths, and the right hippocampus in allocentric coding [18], [19]. There are also important gender differences in spatial cognition [20]. Men prefer strategies with global, allocentric reference frames, whereas women more often make use of route and kinesthaetic strategies and local landmarks [21]–[25].

In the present study we investigate a hypothesis about the brain mechanisms involved in a third mode of presentation in virtual navigation which uses an *oblique* or *slanted* perspective. Modern navigation aids often use this slanted perspective. We hypothesized that this empirical choice allows the human brain to store spatial information very efficiently, making parallel use of the two main cognitive strategies (egocentric and allocentric) which are known to be used for navigation and spatial memory. This parallel encoding could allow navigators to use either of these types of information during recall depending on their cognitive capacities, task and context. We investigated the brain areas activated during the learning of a virtual city from a slanted perspective view, a topic that to our knowledge has never previously been studied. It provides the observer with a compromise between route and survey perspectives, since it involves both information about landmarks as in route perspective and geometric information as in survey perspective. In order to test our hypothesis, we developed a new paradigm for testing performance, in which we asked observers to find a shortcut and compared the performance and brain activity while subjects found a shortcut after learning from the three different perspectives (route, slanted, survey).

In order to obtain a clear understanding of the mechanisms involved we also tried to dissociate encoding and recall. Only two studies have directly investigated brain activation during route and survey encoding. Shelton and Gabrieli [26] were the first to suggest that route and survey encoding recruit a common network of brain areas. They found that survey encoding recruits a subset of areas involved in route encoding, but with greater activation in inferior temporal and posterior superior parietal cortex. They also showed that route encoding recruited regions that were not activated by survey encoding, including medial temporal lobe structures, anterior superior parietal cortex, and postcentral gyrus. However, these results were contradicted and criticized by Blanch et al. [27], who argued that Shelton and Gabrielli [26] did not use a baseline condition, so that the differences they found may in fact reflect visual aspects of the task stimuli. In their study, Blanch et al. [27] compared the substrates of path learning from different perspectives (route and survey) and observed that many areas are recruited in route learning from both perspectives, such as parahippocampus, precuneus, posterior cingulate gyrus, and middle frontal gyrus. However, they also showed that survey perspective activates the superior and middle temporal gyri and the angular gyrus, which are not activated in the route perspective.

In addition, different brain areas are involved in the encoding and recall of small- and large-scale environments [28]. Foo et al. [29] argued that coarse, possibly nonmetric, spatial knowledge can be derived from route perspective, but that it is limited by the resolution and biases of the human path integration system. According to Foo et al. [29], in a shortcut task performed from route perspective, humans do not build up survey knowledge on the basis of path integration but instead use coarse spatial knowledge. In contrast, when survey perspectives are provided, survey (map-like) knowledge should be used directly to accurately derive shortcuts.

We hypothesized that slanted perspective that is a hybrid point of view between route and survey perspectives should elicit activations of brain areas that are also specifically activated by the route and survey perspectives. We expected that route and slanted perspectives would activate the medial temporal lobe structures, anterior parietal cortex, and postcentral gyrus since these structures were reported to be specific to route encoding [26]. We also expected that survey and slanted perspectives would elicit activations of the superior and the middle temporal gyri and the angular gyrus since these brain areas were considered to be specific to survey encoding [27].

Furthermore, Shelton and Gabrielli [26] reported that route and survey perspectives recruited common brain areas including inferior temporal cortex, the posterior and superior parietal cortex but with greater activations in survey perspective than in route one. Slanted perspective provides information closed to route perspective (i.e. landmarks) but also closed to survey perspective (i.e. the global configuration). Nevertheless, these information are in-between route and survey information (landmarks are less salient in slanted perspective than in route one; the global configuration is less salient in slanted perspective than in survey one). In these conditions, intermediate intensity of activations in brain structures recruited by both route and survey perspectives were possible in slanted perspective.

## Materials and Methods

### Participants

Twenty-six right-handed participants (13 males; age: 18–29 years) were included in this study. All subjects were free of neurological disease and injury and had no abnormality on T1-weighted magnetic resonance imaging (MRI). The local Ethics Committee (Comité de protection des personnes, Ile-de-France; n°2008-A00327-48) approved this study and written informed consent was obtained from each participant.

### Experimental set-up

The virtual environment (developed by ARCHIVIDEO) represented a virtual city which was inspired by the topography and building architecture of Paris, but which was devoid of objects (cars, trees, etc.), traffic signs (traffic lights, zebra crossings, etc.) and human figures for the purposes of the experiment.

This virtual city was displayed on a 19-inch screen using Virtools 4.0 software. Participants controlled their navigation within the virtual environment by manipulating a joystick with their right hand. Possible movements included going forward, going backward, turning left, turning right and stopping. During exploration, navigation speed could not exceed 10 m/s. None of the subjects had seen the virtual city before the experiment.

### Procedures

#### Experimental conditions

In our experiment, subjects were asked to encode a path (passive navigation) and based on this encoding to navigate to the same destination by means of a shortcut (active navigation). These 2 tasks (encoding and shortcut) were performed from three different perspectives (route, slanted, survey). The 6 resulting experimental conditions were matched with 6 baseline conditions (passive and active navigation for each of the 3 perspectives) in order to subtract any brain activations not directly relevant to the activity of storage, recall or processing of navigational information.

#### Encoding task

**Figure 1:**
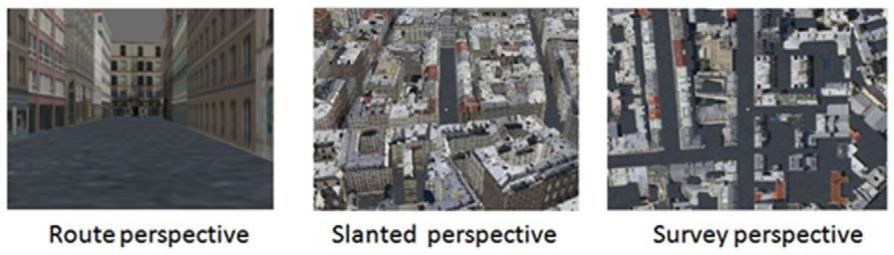
Examples of route, slanted, and survey perspective views.

Participants were shown a 40-s movie of navigation within the virtual city on the screen and asked to learn the path from the starting point to the goal. The speed of the navigation movie was set to 1 m/s, which corresponds to walking speed. The point of view used in the movie could either be a route, survey or slanted perspective (Figure 1). The route perspective corresponded to a first-person view of the city (height of 1.70 virtual metres). The survey perspective was an aerial point of view that was perpendicular to the ground. The height used for the survey perspective was determined in a preliminary experiment with 10 subjects which revealed that they succeeded better on a shortcut-finding task with a height of 200 virtual metres than 170 or 230 virtual metres (all p<0.01). The slanted perspective, a compromise between the first two perspectives, was set to an oblique angle of view of 45° at 170 virtual metres from the floor. Angle and height were experimentally determined in a preliminary experiment using three angles of view (35°, 45°, 65°), determined by a pre-test, and 3 heights (170, 200, 230 virtual metres). Twelve subjects who had not participated in the previous pre-test were asked to perform a shortcut-finding task after passively viewing a path in the 3 angle * 3 height conditions. Analysis of percentage success in the various conditions showed that the performance was statistically best in the condition with the 45° angle of view and 170-virtual-metre height compared to the other conditions (all p<0.01). A compromise between height and angle was thus needed to see the floor and encode the path optimally.

For the main experiment, we designed a set of 10 paths (distinct configurations but same length) which was identical for the three perspectives, in order to ensure that all the perspectives were equivalent in terms of difficulty of path configuration. In order optimize the comparability of the 3 perspectives, all included turns [30].

#### Shortcut task

**Figure 2:**
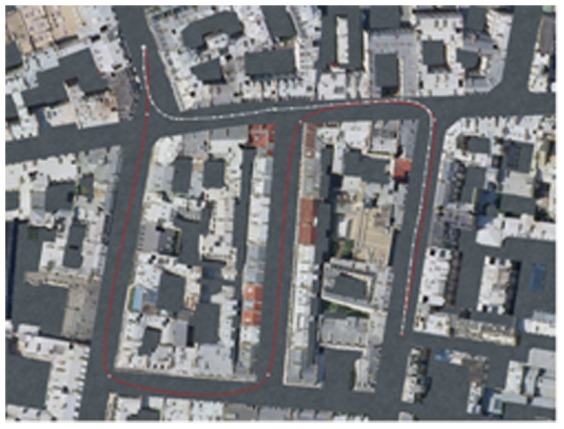
Example (from survey perspective) of a path from the encoding condition (in red) and of a correct shortcut that a subject produced for the same path (in white).

Immediately after the navigation movie, participants were required to navigate to the goal from the starting point using a joystick with the right hand while taking a shortcut—i.e., a shorter path than the one that they had experienced during the encoding task. The participants were told that when multiple shortcuts were available, they should take the shortest one. It is worth noting that the point of view was always identical within a given encoding-shortcut sequence. For example, an encoding task from a survey perspective was necessarily followed by the performance of a shortcut task also from a survey perspective. The shortcut task was time-limited (50 s). When the goal was reached or time ran out, the fixation cross for the following (baseline) condition was automatically presented and the following trial initiated. If necessary, participants could also decide to press a key in order to abandon the current trial and to proceed to the following one. This allowed us to ensure that the subjects were still performing the task until the following task was delivered or the key was pressed. Response accuracy, travel time and distance travelled were recorded using Virtools 4.0 software. Response accuracy corresponded to the proportion of trials that were not aborted and for which the shortcut path was shorter than the encoding path. Travel time corresponded to the elapsed time between the beginning of the trial and the moment when the subject arrived at the destination. Only the travel time on correct trials (not aborted and where the path in the shortcut task was shorter than the one taken during the encoding) was considered in the analysis. An example of a correct trial is presented in Figure 2. The distance travelled corresponded to the length (in virtual metres) of the correct shortcut paths.

#### Baseline conditions

In order to subtract any brain activity that was not directly relevant, we asked subjects to perform some control tasks. As mentioned in the introduction, Blanch et al. [27] argued that the baseline condition is crucial in order to isolate the parts of the brain specifically recruited in learning from different perspectives. Indeed, the brain areas they reported for route and survey perspective encoding contradicted the results of Shelton and Gabrielli [26], who compared the two perspectives directly (without baseline conditions). In order to control for possible effects of the visual differences between conditions as suggested by Blanch et al. [27], we used specific baseline conditions for each perspective (route, slanted and survey) and both tasks (encoding versus shortcut-finding).

#### A passive navigation task was used as a baseline condition for the encoding tasks

Participants were presented a 40-s movie of navigation within the virtual city on the screen and asked to passively watch the path from the start point to the goal. The point of view used in the movie could be a route, survey or slanted perspective. Note that the paths were different from those used in the experimental conditions. This control task reference was intended to evoke similar visual activations as in the encoding task but without activating higher-level regions specifically involved in path encoding.

#### An active navigation task was used as baseline for the shortcut-finding task

Participants had to follow a line plotted on the ground within the virtual city using the joystick for 40 s. The point of view used during exploration could either be a route, survey or slanted perspective. Note that here again the paths followed by participants were different from those used in the experimental conditions. This reference was expected to activate similar visual and motor areas as the shortcut task but without involving regions underlying navigational strategies.

#### Scanning session (Figure 3)

**Figure 3:**
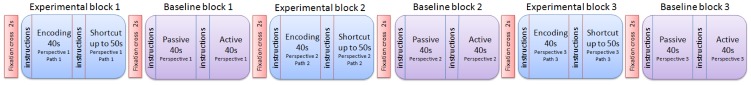
Schematic representation of events during an fMRI scanning session.

Each participant performed 10 scanning sessions, each including 6 pairs of blocks: 3 pairs of experimental blocks (encoding block + shortcut block), and 3 pairs of baseline blocks (passive navigation block + active navigation block). Each block began with the presentation of a fixation cross for 2 seconds. The 3 pairs of experimental blocks (encoding block + shortcut block) alternated with the 3 pairs of navigation baseline blocks (Figure 3). The perspectives in the paired encoding and shortcut blocks and in the following paired baseline blocks were always identical. In each scanning session, the 3 perspectives (route, slanted and survey) were presented, and the order of perspectives was pseudo-randomly determined. The order of the 3 perspectives was drawn at random between the 6 existing orders without replacement for the six first sessions and the order of perspectives in the 4 last sessions was again drawn at random between the 6 possible orders without replacement. Furthermore, in order to control for the difficulty of path configuration, we used the same path in the 3 perspectives. Because of the possibility of a learning effect, we counterbalanced the order of the 3 perspectives for a given path between subjects (e.g. Subject 1: path 1, perspective 1, 2, 3; Subject 2: path 1; perspective 3, 1, 2). Furthermore, a given path was used for the second time only when all 10 paths had been presented from the first perspective view. For example, if path 1 was presented from a given perspective during the first scanning session, then this path was used for the second perspective during the 4^th^ scanning session and during the 7^th^ scanning session for the last perspective. Post-experimental debriefing confirmed that the subjects did not realize that the same paths had been presented from different perspectives. Each task was cued by a short instruction displayed on the screen informing the subject of the nature of the following task. Immediately prior to the first scanning session, subjects underwent a training phase consisting of one session that was similar to the sessions performed in the MRI scanner.

### Behavioral data analysis

No behavioural measures could be recorded during the encoding task. We measured performance on the shortcut task: *response accuracy* (number of non-aborted trials where the path in the shortcut task was shorter than the one taken in the encoding task), *travel time* (for correct shortcuts in the virtual world), and *distance travelled* (for correct trials, in virtual metres).

Analyses of variance (ANOVAs) with perspective (route, slanted, survey) as a within-subjects factor and gender (male, female) as a between-subjects factor were performed on these measurements. Tukey's post hoc analyses were also computed when needed. The statistical threshold was set to α = 0.05.

### fMRI data acquisition

Gradient echoplanar images sensitive to brain oxygen-level-dependent (BOLD) signal were acquired with a 3-Tesla Siemens body scanner at Neurospin Center (Saclay, France). Each volume included 40 axial slices (thickness = 3 mm, TR = 2400 ms, TE = 30 ms, flip angle = 81°, FOV = 192 mm, matrix = 64×64). Participants wore earplugs to attenuate scanner noise, and padding was used to reduce head movements. A standard shimming procedure was performed before each scanning session to minimize inhomogeneities in the static magnetic field. In each session, a maximum of 190 functional volumes was acquired. The first 2 volumes were discarded to reach equilibrium. T1-weighted images were also acquired for anatomical localization (3D-MPRAGE, 160 sagittal slices, thickness = 1.1 mm, TR = 2300 ms, TE = 2.98 ms, flip angle = 9°, FOV = 256 mm, matrix = 256×256).

### fMRI data processing

Imaging data were processed using SPM5 software (Wellcome Department of Imaging Neuroscience, London, U.K.). Processing included correction for differences in slice acquisition time using the first slice as the reference, spatial realignment to the first volume, spatial normalization into the Montreal Neurological Institute (MNI) stereotaxic space [31] and spatial smoothing using an isotropic Gaussian kernel with a full width at half maximum (FWHM) of 6 mm. A high-pass filter with a cut-off period of 400 s was also applied to remove low-frequency drifts from the data.

### fMRI data analysis

Statistical analyses were carried out using a two-stage generalized linear model (GLM). Task-specific effects were modelled separately for each participant. Regressor functions were constructed by convolving time series for each condition type with a standard hemodynamic response function. Fourteen regressors were defined: 6 regressors for each experimental condition (encoding/route, encoding/slanted, encoding/survey, shortcut/route, shortcut/slanted and shortcut/survey) and 6 regressors for each baseline condition (passive nav./route, passive nav./slanted, passive nav./survey, active nav./route, active nav./slanted and active nav./survey). Six additional covariates corresponding to realignment parameters were also included in order to capture residual movement-related artifacts.

In the second stage of analysis, the results were analyzed at the group level. An ANOVA was performed with gender (males, females) as a between-subjects factor and task (encoding, shortcut) and perspective (route, slanted, survey) as within-subjects factors. Images of parameter estimates obtained at the subject level for the 6 experimental conditions relative to baseline (passive navigation baseline for the 3 encoding conditions and active navigation baseline for the 3 shortcut ones) were included. A non-sphericity correction was applied for variance differences across conditions or subjects. Variance estimates at the group level thus incorporated appropriately weighted within-subject and between-subject variance effects. In this analysis, the following contrasts were computed:

1. Mean cerebral activations for route, slanted and survey perspectives regardless of gender or task
2. Cerebral activation differences between perspectives regardless of gender or task: route minus slanted, slanted minus route, route minus survey, survey minus route, slanted minus survey, and survey minus slanted.
3. Cerebral differences between genders regardless of task or perspective: males minus females and females minus males.
4. Cerebral differences between tasks regardless of gender or perspective: encoding minus shortcut and shortcut minus encoding.
5. Interactions between gender, task and perspective.

For all statistical maps, we reported activations that survived a family-wise error (FWE)-corrected threshold of α = 0.05 with a minimum cluster extent of 10 contiguous voxels. For all contrasts except the first (mean activations for the three perspectives), the volume of comparison was restricted to significant voxels in subtractions of the relevant baseline from route, slanted, survey, male, female, encoding and finally shortcut activations. For the reported contrasts we used a threshold of α = 0.05, corrected for multiple comparisons. Anatomical localization was performed using Anatomical Automatic Labelling (AAL) [32].

## Results

### Behavioural results (Table 1)

**Table 1:** Response accuracy (in percentage of correct trials), travel time (in seconds) and distance travelled (in virtual metres) measured during the shortcut-finding task are presented for the two genders (women, men) and the three perspectives (route, slanted, survey).

		Perspective view		
	Gender	*Route*	*Slanted*	*Survey*
**Response accuracy** (%)	*women*	48.4±25%	80.8±14%	86.2±12%
	*men*	63.1±18%	89.2±13%	92.3±6%
**Travel time** (seconds)	*women*	28.9±3.3s	30.1±2.9s	28.8±2.0s
	*men*	27.6±4.2s	26.9±3s	25.8±2.5s
**Distance travelled** (virtual metres)	*women*	20.5±2 vm	21.5±1.1vm	20.0±0.7 vm
	*men*	20.7±2.1 vm	20.1±1.2 vm	20.1±0.7 vm

#### Response accuracy

For response accuracy (proportion of successful shortcuts) the ANOVA revealed an effect of perspective (F(2,48) = 39.01; P<0.0001). Tukey's post hoc tests showed that response accuracy was significantly higher for slanted (85.0±13.9%) and survey (89.2±9.8%) perspectives than for route perspective (55.8±22.8%, all p<0.01). Response accuracy was also slightly lower for slanted than for survey perspective (p<0.05). These results indicate that slanted perspective induced response accuracy intermediate between survey and route perspectives, although performance in this condition was closer to the survey than to the route condition. The analysis also revealed an effect of gender (F(1,24) = 5.89; P = 0.02): men (81.5±18.4%) slightly outperformed women (71.8±24.5%) in finding shortcuts. However standard deviations were high enough to indicate a large overlap in this ability. No interaction was found between gender and perspective (F(2,48) = 0.56; p = 0.57).

#### Travel time

The results showed that the overall range of travel time varied from 26.9±3 seconds for men in survey perspective to 30.1±2.9 seconds for women in route perspective (table 1). Statistical analysis (ANOVA) of correct trials (time elapsed to reach the final position for correct shortcuts) revealed a gender effect (F(1,23) = 5.13; P = 0.03) but no perspective effect (F(2,46) = 1.80; P = 0.18) and no interaction between gender and perspective (F(2,46) = 1.28; P = 0.29).

Men (26.8±3.3 s) performed the task slightly faster than women (29.2±3.1 s).

#### Travelled distance

The analysis (ANOVA) of distance travelled on correct shortcuts did not reveal an effect of gender (F(1,23) = 0.001; p = 0.98) or perspective (F(2,46) = 1.77; p = 0.18) or an interaction between gender and perspective (F(2,46) = 0.14; p = 0.86). The observed difference in travel time between women and men, mentioned above, was not due to longer paths, since no difference in traveled distance was observed between women and men. A qualitative investigation of the kinematics (shape of trajectories, stops at crossroads) of women's and men's paths revealed that women hesitated more than men at intersections.

Taken together, the behavioural results revealed an effect of gender on shortcut-finding performance (with men outperforming women) for all perspective views. Furthermore, we observed that both slanted and survey perspectives led to optimal performance, while route perspective was associated with lower performance.

### fMRI results

#### Mean activations with route, slanted and survey perspectives (Figure 4)

**Figure 4:**
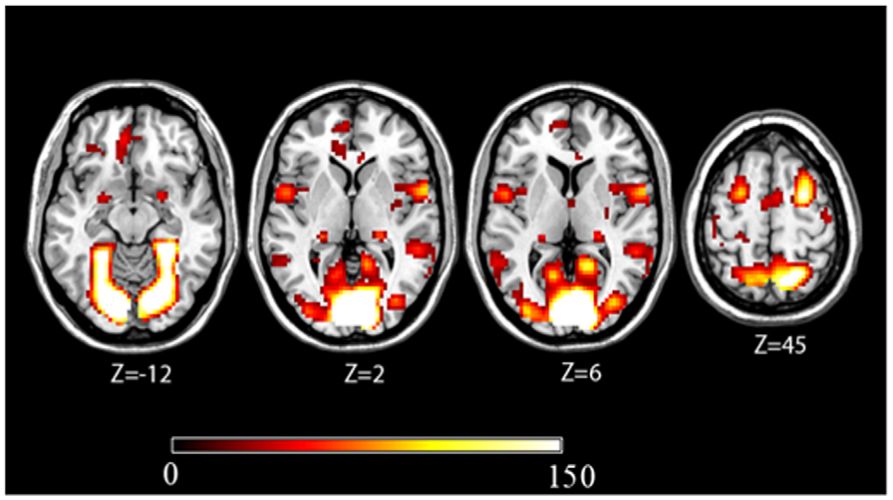
Cerebral activations observed for all experimental conditions (encoding and shortcut for the 3 perspectives (minus their baselines)). The statistical threshold was set at (FWE-corrected) p<0.05 and cluster size at 10 voxels.

When pooling all activations irrespective of gender or task, the three perspectives activated a large bilateral neural network encompassing frontal, parietal, occipito-temporal and cerebellar cortices during both encoding and shortcut tasks compared to baseline (Table 2). In the frontal lobe, bilateral activations were detected in the frontal eye fields, extending to the right middle frontal gyrus and the left superior medial frontal gyrus. In the parietal lobe, we observed bilateral activation in the superior parietal lobule at the junction with the intraparietal sulcus, and in the precuneus. Bilateral occipito-temporal activations were found in the parahippocampal place area, retrosplenial cortex, and superior occipital gyrus. Left-side activation was also observed in the fusiform gyrus and the cuneus. In the cerebellar cortex, we found activation in the vermis.

**Table 2:** Mean activations in route, slanted and survey perspective conditions during encoding and shortcut tasks compared to baseline.

Mean activations in route, slanted and survey perspective conditions						
Anatomical region	N voxels	MNI coordinates			p(FWE cor.)	Z value
		X	Y	Z		
	6926					
*Occipito-temporal cortex*						
R mid. occipital gyrus		30	−81	21	<0.001	>8
L mid. occipital gyrus		−27	−87	21	<0.001	>8
R lingual/post. parahippocampal gyrus		27	−48	−6	<0.001	>8
L lingual/post. parahippocampal gyrus		−27	−48	−6	<0.001	>8
L sup. occipital gyrus		−24	−90	24	<0.001	>8
R sup. occipital gyrus		18	−93	21	<0.001	>8
R calcarine/occipito-parietal sulcus		18	−54	18	<0.001	>8
L calcarine/occipito-parietal sulcus		−18	−60	18	<0.001	>8
L fusiform gyrus		−27	−57	−9	<0.001	>8
L cuneus		−15	−72	33	<0.001	>8
*Parietal cortex*						
R sup. parietal lobule/intraparietal sulcus		21	−75	51	<0.001	>8
L sup. parietal lobule/intraparietal sulcus		−18	−72	45	<0.001	>8
R sup. parietal lobule/precuneus		15	−63	60	<0.001	>8
L precuneus		−9	−78	45	<0.001	>8
*Cerebellar cortex*						
Vermis		0	−75	−27	<0.001	>8
*Frontal cortex*						
R sup. frontal/precentral sulcus	366	27	3	60	<0.001	>8
R mid. frontal gyrus		27	30	42	<0.001	5.95
L sup. frontal/precentral sulcus	192	−21	3	57	<0.001	>8
L sup. medial frontal gyrus	17	0	30	42	<0.001	5.64
*Cerebellar cortex*						
Vermis	34	0	−57	−36	<0.001	>8

The statistical threshold was set at (FWE-corrected) p<0.05 and cluster size at 10 voxels (L: left; R: right).

#### Effect of perspective regardless of gender and task (encoding/shortcut task)

**Figure 5:**
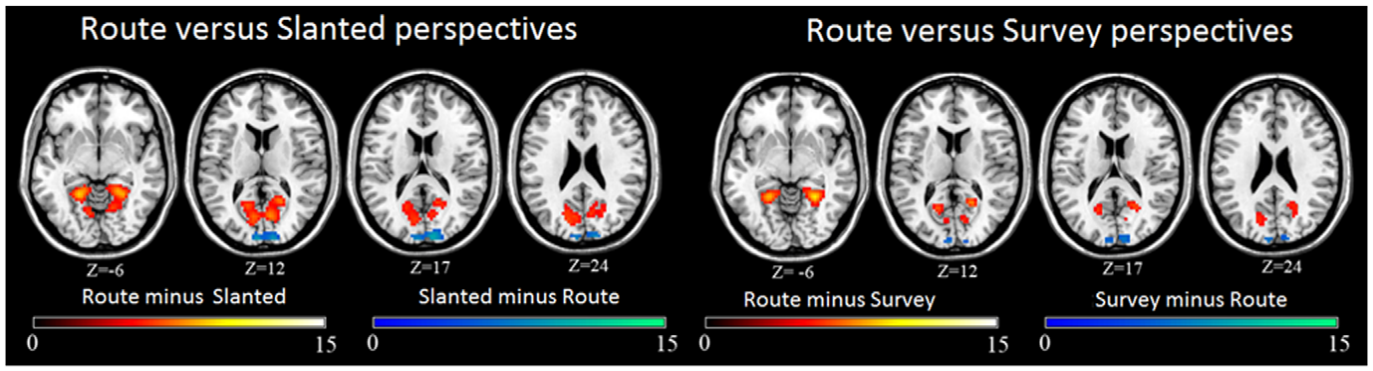
Cerebral differences between route (minus its baseline), slanted (minus its baseline) and survey (minus its baseline) perspectives during both encoding and shortcut tasks. The statistical threshold was set at (FWE-corrected) p<0.05 and cluster size at 10 voxels.

We first analyzed the effect of perspective independently of gender and task. Because we were especially interested in the slanted perspective, we compared it with the two other perspectives. We observed that, in comparison to the route perspective, the slanted perspective (route minus its baseline subtracted from slanted minus its baseline; Table 3 and Figure 5) was only specifically associated with occipital activations in the cuneus bilaterally and the left calcarine sulcus. In contrast, we observed that in the reverse comparison (slanted minus its baseline subtracted from route minus its baseline; Table 3 and Figure 5) route perspective was specifically associated with bilateral occipito-temporal activations in the parahippocampal place area and retrosplenial cortex, extending to left superior occipital gyrus and right fusiform gyrus (Table 3). Activations were also found in the right precuneus and the left cerebellum. We observed a similar pattern of results when we contrasted route and survey perspectives. Compared to survey perspective, route perspective (survey minus its baseline subtracted from route minus its baseline; Table 3 and Figure 5) was specifically associated with bilateral occipito-temporal activations in the parahippocampal place area, retrosplenial cortex and the fusiform gyrus (Table 4). Activations were also noted in the left superior occipital gyrus and the right precuneus. We also observed that compared to route perspective, survey perspective (route minus its baseline subtracted from survey minus its baseline; Table 4 and Figure 5) was only specifically associated with occipital activations in the cuneus bilaterally and in the left calcarine sulcus.

**Table 3:** Cerebral differences between route and slanted perspectives during both encoding and shortcut tasks compared to baseline.

Route perspective minus slanted perspective						
Anatomical region	N voxels	MNI coordinates			p(FWE cor.)	Z value
		X	Y	Z		
	1325					
*Occipito-temporal cortex*						
L lingual/post. parahippocampal gyrus		−21	−48	−6	<0.001	>8
R lingual/post. parahippocampal gyrus		24	−48	−3	<0.001	>8
L calcarine/occipito-parietal sulcus		−12	−60	6	<0.001	7.51
R calcarine/occipito-parietal sulcus		18	−51	6	<0.001	7.31
L sup. occipital gyrus		−15	−75	24	<0.001	6.15
L cuneus		−9	−72	24	<0.001	6.08
R cuneus		18	−72	33	<0.001	6.04
R fusiform gyrus		36	−48	−18	<0.001	6.08
*Parietal cortex*						
R precuneus		24	−60	21	<0.001	5.73
*Cerebellar cortex*						
L cerebellum	30	−6	−78	−24	<0.001	5.45

Slanted perspective minus route perspective						
Anatomical region	N voxels	MNI coordinates			p(FWE cor.)	Z value
		X	Y	Z		
*Occipital cortex*						
R cuneus	219	9	−93	18	<0.001	>8
L cuneus		−6	−96	21	<0.001	6.73
L calcarine sulcus		−3	−96	12	<0.001	7.73

The statistical threshold was set at (FWE-corrected) p<0.05 and cluster size at 10 voxels (L: left; R: right).

**Table 4:** Cerebral differences between route and survey perspectives during both encoding and shortcut tasks compared to baseline.

Route perspective minus survey perspective						
Anatomical region	N voxels	MNI coordinates			p(FWE cor.)	Z value
		X	Y	Z		
	340					
*Occipital cortex*						
R lingual gyrus		27	−48	−6	<0.001	>8
R calcarine/occipito-parietal sulcus		18	−51	9	<0.001	7.08
R cuneus		18	−72	33	<0.001	5.78
*Parietal cortex*						
R precuneus		24	−60	21	<0.001	6.38
*Temporal cortex*						
R post. parahippocampal gyrus		18	−36	−12	<0.001	6.02
R fusiform gyrus		30	−57	−12	<0.001	5.75
*Occipito-temporal cortex*						
L lingual/post. parahippocampal gyrus	266	−21	−48	−6	<0.001	>8
L calcarine/occipito-parietal sulcus		−15	−60	9	<0.001	6.94
L sup. occipital gyrus		−15	−72	30	<0.001	5.71
L cuneus		−12	−75	33	<0.001	5.69
L fusiform gyrus		−30	−63	−15	<0.007	5.11
R lingual gyrus	44	9	−69	3	<0.001	5.69
R calcarine sulcus		12	−72	12	<0.001	5.5
L lingual gyrus	13	−9	−69	0	<0.012	5.01
L calcarine sulcus		−9	−72	9	<0.024	4.84

Survey perspective minus route perspective						
Anatomical region	N voxels	MNI coordinates			p(FWE cor.)	Z value
		X	Y	Z		
*Occipital cortex*						
R cuneus	41	9	−93	18	<0.001	6.52
L calcarine sulcus	35	−3	−96	12	<0.001	6.16
L cuneus		−6	−96	24	<0.001	5.99

The statistical threshold was set at (FWE-corrected) p < 0.05 and cluster size at 10 voxels (L: left; R: right).

These results confirm that many of the same brain areas were used to encode and navigate from both route and survey perspectives, but that the route perspective recruited extra brain areas. Furthermore, the brain activations associated with the slanted perspective were similar to those associated to the survey perspective. Direct comparison of survey and slanted perspective activations (survey minus its baseline subtracted from slanted minus its baseline, and vice versa) revealed that no brain structures were more activated in one than in the other of these two perspectives.

#### Gender effect

The gender effect observed in behavioural performance was not reflected in any sizeable differences in brain activation. When male and female brain activations were compared, only a small focus of activation in the right calcarine sulcus was observed (males minus females) (Table 5).

**Table 5:** Cerebral differences between males and females during both encoding and shortcut tasks compared to baseline regardless of perspective.

Males minus females						
Anatomical region	N voxels	MNI coordinates			p(FWE cor.)	Z value
		X	Y	Z		
*Occipital cortex*						
R calcarine sulcus	27	6	−84	9	<0.001	6.3

Females minus males						
Anatomical region	N voxels	MNI coordinates			p(FWE cor.)	Z value
		X	Y	Z		
*No suprathreshold clusters*						

The statistical threshold was set at (FWE-corrected) p<0.05 and cluster size at 10 voxels (L: left; R: right).

#### Effect of task (encoding vs. shortcut) regardless of gender and perspective

**Figure 6:**
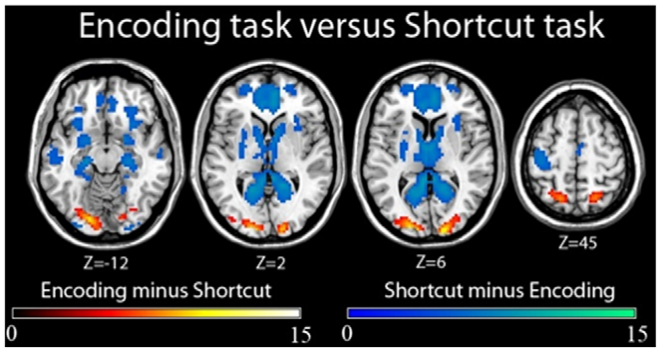
Cerebral differences between encoding and shortcut tasks regardless of perspective. The statistical threshold was set at (FWE-corrected) p<0.05 and cluster size at 10 voxels.

When we analyzed the effect of task independently of gender and perspective, we observed that compared to the shortcut task, the encoding task (encoding minus shortcut; Figure 6) was specifically associated with occipital activations in the superior and middle occipital gyrus, in the lingual gyrus bilaterally, and in the right cuneus and left calcarine sulcus (Table 6). Bilateral parietal activations were also found in the inferior and superior parietal lobules at the junction with the intraparietal sulcus. In contrast, we observed that compared to the encoding task, the shortcut task (shortcut minus encoding; figure 6) elicited activity in a large network encompassing frontal, paralimbic, occipital, parietal, temporal cortices and subcortical areas (Table 6). In the frontal and paralimbic cortices, we detected bilateral activations in the posterior, middle and anterior cingulate cortex, the insula, and the superior frontal gyrus. Activity was also found in the right medial orbital frontal gyrus and the right inferior frontal gyrus (pars orbitaris). In the medial temporal lobe, we observed bilateral activations in the hippocampus at the junction with the parahippocampal gyrus. Subcortical activity was also observed in the bilateral thalamus, the right putamen and the right caudate nucleus. Parietal activations included the precuneus at the intersection with the occipito-parietal sulcus bilaterally, as well as right angular gyrus. In the occipital cortex, activations were noted in the lingual gyrus bilaterally and in right inferior occipital gyrus, left cuneus and right calcarine sulcus.

**Table 6:** Cerebral differences between encoding and shortcut tasks regardless of perspective.

Encoding task minus shortcut task						
Anatomical region	N voxels	MNI coordinates			p(FWE cor.)	Z value
		X	Y	Z		
*Occipital cortex*						
R cuneus	291	18	−96	9	<0.001	>8
R sup. occipital gyrus		24	−93	15	<0.001	>8
R mid. occipital gyrus		30	−90	12	<0.001	>8
R lingual gyrus		15	−87	−6	<0.001	7.38
L lingual gyrus	283	−18	−87	−9	<0.001	>8
L calcarine sulcus		−9	−96	−6	<0.001	>8
L mid. occipital gyrus		−27	−93	12	<0.001	>8
L sup. occipital gyrus		−12	−96	6	<0.001	7.27
*Parietal cortex*						
L sup. parietal lobule/intraparietal sulcus	113	−18	−63	57	<0.001	7.51
L inf. parietal lobule		−39	−42	48	<0.001	5.97
R sup. parietal lobule/intraparietal sulcus	60	18	−63	60	<0.001	7
R inf. parietal lobule	15	39	−45	54	<0.005	5.18

Shortcut task minus encoding task						
Anatomical region	N voxels	MNI coordinates			p(FWE cor.)	Z value
		X	Y	Z		
*Occipital cortex*						
R inf. occipital gyrus	13	27	−96	−6	<0.001	>8
						
	2214					
*Paralimbic cortex*						
L post. cingulate cortex		−3	−45	24	<0.001	>8
R post. cingulate cortex		6	−48	27	<0.001	>8
L mid. cingulate cortex		−3	−36	39	<0.001	>8
R mid. cingulate cortex		9	−24	42	<0.001	7.71
*Parietal cortex*						
L precuneus/occipito-parietal sulcus		−6	−54	12	<0.001	>8
R precuneus/occipito-parietal sulcus		6	−69	30	<0.001	>8
*Occipital cortex*						
L cuneus		−6	−69	27	<0.001	>8
L lingual gyrus		−18	−54	3	<0.001	>8
R lingual gyrus		6	−42	6	<0.001	7.76
R calcarine sulcus		9	−54	12	<0.001	7.65
*Temporal cortex*						
L hippocampus/parahippocampal gyrus		−21	−21	−15	<0.001	>8
R hippocampus/parahippocampal gyrus	51	24	−21	−15	<0.001	7.75
*Frontal cortex/Paralimbic cortex*						
L ant. cingulate cortex	454	−6	36	12	<0.001	>8
R ant. cingulate cortex		6	30	27	<0.001	>8
R medial orbital frontal gyrus		6	42	−6	<0.001	>8
L insula	74	−27	18	−9	<0.001	7.02
L mid. cingulate cortex	24	−6	−21	45	<0.001	6.91
L sup. frontal gyrus	44	−21	57	6	<0.001	6.28
R mid. cingulate cortex	27	3	9	30	<0.001	6.22
R ant. cingulate cortex		3	18	21	<0.001	5.74
R sup. frontal gyrus	38	24	57	6	0.001	5.61
*Subcortical areas*						
L thalamus	54	−6	−6	6	<0.001	7.78
R thalamus	40	6	−12	12	<0.001	7.55
*Subcortical areas*						
R putamen		24	18	−6	<0.001	6.79
R caudate nucleus		12	15	0	<0.001	6.64
*Frontal cortex/Paralimbic cortex*						
R insula		30	18	−12	<0.001	6.46
R frontal gyrus pars orbitaris		33	33	−6	<0.001	5.82
*Parietal cortex*						
R angular gyrus	39	45	−69	42	<0.001	6.56

The statistical threshold was set at (FWE-corrected) p<0.05 and cluster size at 10 voxels (L: left; R: right).

#### Interactions between gender, task and perspective

**Figure 7:**
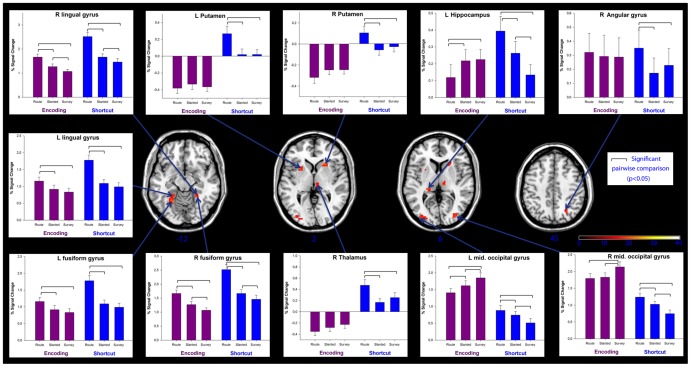
Cerebral differences observed in the interaction between task (encoding, shortcut) and perspective (route, slanted, and survey minus their baselines). The statistical threshold was set at (FWE-corrected) p<0.05 and cluster size at 10 voxels. The percentage signal change (scaled beta weight) for each condition is given for the regions of interest. Pairwise comparisons were performed using Tukey HSD tests. Error bars reflect the standard error of the mean.

We first analyzed the interaction between the three variables: Gender (2) x Task (2) x Perspective (3). This test for second-order interaction effect did not reveal any brain activations. Results were similar for Gender (2) x Task (2) and Gender (2) x Perspective (2) interactions. The only analysis of a first-order interaction that revealed a statistical cluster of cerebral activations was the interaction between Task (2) and Perspective (3). This interaction was specifically associated with bilateral occipital activations in the middle occipital gyrus and the lingual gyrus as well as in the fusiform gyrus (Table 7). Bilateral activation of the angular gyrus was also found. In the medial temporal lobe, we observed activation in the left hippocampus. Subcortical activity was also observed in the right thalamus and bilaterally in the putamen. In order to analyze these interactions, each of the activated clusters was classified as a region of interest (ROI). For each ROI, mean beta weights for the two tasks and the three perspectives were extracted for each participant, scaled to reflect percent signal change. Separate ANOVAs were conducted for individual ROIs, with task and perspective as within-subjects factors. When an interaction between the two factors was observed, the interaction was examined in more detail using Tukey Honestly Significant Difference (HSD) tests. Interactions were found for all 11 clusters of voxels (all p<0.001). This post hoc examination of the interaction (Figure 7) revealed that the percentage of signal change was higher in route than in slanted and survey conditions for both encoding and shortcut tasks in the left lingual gyrus and the left fusiform gyrus (all p<0.05). These results demonstrate that the slanted and survey perspectives induced similar brain changes in these ROI during both encoding and shortcut tasks. This result was also observed in the bilateral putamen, the thalamus and the right angular cortex for the shortcut task (all p<0.05), but no difference was found for the encoding task (all p>0.13).

**Table 7:** Cerebral differences between males and females during both encoding and shortcut tasks compared to baseline regardless of perspective.

Interaction task (Encoding vs shortcut) * perspective (route vs slanted vs survey)						
Anatomical region	N voxels	MNI coordinates			p(FWE cor.)	Z value
		X	Y	Z		
*Occipital cortex*						
R Mid Occipital	77	30	−78	18	<0,001	5,78
L Mid Occipital	63	−27	−90	9	<0,001	5,47
*Occipito-temporal cortex*						
L Lingual	38	−24	−57	−12	<0,001	5,61
R Lingual	15	30	−48	−9	<0,009	5,1
R Fusiform		24	−42	−12	<0,012	5,03
L Fusiform		−24	−45	−12	<0,005	5,21
*Temporal cortex*						
L Hippocampus	14	−19	−34	6	<0,001	5,87
*Parietal cortex*						
R Angular	19	27	−63	45	<0,003	5,32
*Subcortical areas*						
L Putamen	25	−27	12	3	<0,001	5,53
R Putamen	18	24	21	3	<0,001	5,73
R Thalamus	20	12	−15	15	<0,005	5,2

The statistical threshold was set at (FWE-corrected) p<0.05 and cluster size at 10 voxels (L: left; R: right).

Interestingly, during encoding, percentage signal change was lower in the left hippocampus in the route perspective than both slanted and survey perspectives (all p<0.05), with no difference between the two last perspectives (p = 0.86). In contrast, during the shortcut task, the percentage of signal change was higher in route perspective than slanted and survey conditions (all p<0.05). Furthermore, the percentage of signal change was higher in the slanted than in the survey perspective condition (p<0.05). These results suggest that the slanted perspective may induce an intermediate change in brain activation in left hippocampus during a shortcut task. This intermediate change in brain activation during encoding in slanted perspective compared to route and survey perspectives was also found in right lingual gyrus, right fusiform gyrus and right middle occipital gyrus (all p<0.05). On the shortcut task too, slanted perspective induced an intermediate change in brain activity in the right fusiform gyrus and in bilateral middle occipital gyrus. During the shortcut task, the route perspective induced a lower percentage of signal change than the slanted perspective, which in turn induced less signal change than the survey perspective, in these regions of interest (all p<0.05). These results again suggest that the slanted perspective may induce specific changes in brain activity which falls in between those induced by route and survey perspectives.

The results also reveal that during encoding, the route and slanted perspectives induced a similar percentage signal change (p = 0.90) in right middle occipital gyrus, which was lower than the change induced by the survey perspective (all p<0.05). These results suggest that route and slanted perspectives may lead to similar brain activations, different from those that occur in survey perspective.

## Discussion

The aim of this study was to determine how brain activity differs during the encoding of spatial information in route, slanted, and survey perspective navigation in a virtual city, and what neural structures are engaged when subjects have to devise and navigate a shortcut using these three different perspectives.

### Behavioural results

A first important result of our study confirms the difficulty of finding a shortcut after a route perspective encoding, and the advantage of both survey and slanted perspectives in comparison. Indeed, shortcut-finding performance was lower in the route condition than in the slanted and survey conditions. The information provided to the subjects influenced the type of strategy they were able to use to succeed at the task. Route knowledge is a one-dimensional representation of the sequence of landmarks and associated turns along a path, while survey knowledge provides information about spatial relationships between landmarks, including orientations and distances. Survey knowledge provided by both slanted and survey perspectives may include relational information about landmarks or road segments between which direct travel has never occurred. The ability to find novel shortcuts between two points is considered to be characteristic of survey knowledge [1], [33]–[35] and the difficulty of finding a shortcut after a route-perspective encoding might be interpreted as reflecting the difficulty of developing an effective strategy to find shortcuts using only coarse and possibly nonmetric spatial knowledge derived from route-perspective encoding.

The second interesting result is that, as we had hypothesized, subjects performed best at finding shortcuts in slanted and survey perspective views (about 85%). These results demonstrated that the slanted perspective led to accurate performance close to what was observed with the survey perspective. At the behavioural level, the slanted perspective may have provided the information required to produce an effective strategy that is probably closed to the survey one.

A third result is that we observed a clear gender effect as previously predicted [9], [21]–[25], with men outperforming women in several respects. Men produced 10% more successful shortcuts than women, who were more likely either not to find a shortcut before the set time limit (50 s) or to give up on a trial due to spatial disorientation. Men also executed their shortcuts more quickly than women, although the quality of men's and women's shortcuts was similar in terms of distance travelled. These results indicate that men were more confident than women during the shortcut-finding task (fewer hesitations at intersections), suggesting that men may build more efficient cognitive maps than women.

### Brain activations

When mean activations for all subjects and the different tasks were pooled, comparison of brain activity in slanted- and survey-perspective conditions showed no significant differences. This confirmed the behavioural data which suggest that survey and slanted perspectives are about equally useful for path encoding and shortcut finding.

The same analysis, however, showed that route perspective recruited regions that were not activated by survey or slanted perspectives, including parahippocampal gyrus and medial occipito-parietal cortex. The involvement of parahippocampus in detecting environmental landmarks during navigation was suggested by Gahem et al [13] and Mellet et al [14] and has since been widely documented. Parahippocampal gyrus has been shown to be active in the encoding of an environment when salient landmarks are present, but not when landmarks are lacking [36]. A possible explanation is that, in the present study, landmarks were used in the route perspective condition, but not the slanted and survey perspective conditions, to encode the environment and find shortcuts. This also fits with the bilateral parahippocampal activations reported during the mental evocation of salient landmarks [37]. Parahippocampal cortex has also been shown to be engaged during the passive viewing of a local environment as compared to passive viewing of objects [7]. In the same vein, these regions have been found to be involved in the retrieval of spatial relationships between objects [38], [39]. Finally, an involvement of parahippocampus has also been found in egocentric and allocentric distance estimation [16] and in the process of memorizing a goal in a virtual reality maze [19]. Foo et al. [29] suggested that coarse, possibly nonmetric, spatial knowledge can be derived from route perspective, but that it is limited by the resolution and biases of the human path integration system. Accordingly, we suggest that route perspective makes heavier demands on processes for inferring global properties from available local properties than survey or slanted perspectives. The involvement of the parahippocampus, on this view, reflects the reliance of inference of possible shortcuts on spatial relationships between landmarks extracted during the path. This process would mostly be needed in the route perspective condition, since no general layout of the environment is provided in this case. In contrast, when survey-type information is provided in the slanted and survey perspective conditions, this map-like knowledge could be used directly to infer accurate shortcuts.

The analysis of percentage signal change performed on the regions of interest significantly activated in the interaction between perspective and task supported our hypothesis that the slanted perspective essentially functions as a hybrid of route and survey perspectives. Indeed, common changes in brain activity were observed between slanted and survey perspectives in the left lingual gyrus and the left fusiform gyrus, while others were observed between route and slanted perspectives, as in right middle occipital gyrus during encoding. The hybrid nature of the slanted perspective was also attested by the fact that the changes in brain activity observed during encoding and shortcut-finding from this perspective in right lingual gyrus, right fusiform gyrus and left middle occipital gyrus fell in between those seen with route and survey perspectives.

In the present study, median occipito-parietal regions (cuneus and precuneus) were activated in the route perspective condition, and not in the slanted or survey perspective conditions. These occipito-parietal activations could be related to richer and more vivid visual imagery [40] during route navigation in this condition than in slanted or survey conditions. Recent findings from functional imaging in healthy subjects suggest a central role for the precuneus in a wide spectrum of highly integrated tasks, including visuo-spatial imagery, episodic memory retrieval, and self-processing operations, namely first-person perspective-taking and the experience of agency (for a review see [41]). The role played by the precuneus remains unclear, but its strong involvement in first-person navigation was recently confirmed by a neuropsychological study [42] involving patients with amnestic mild cognitive impairment. Smaller volume of right precuneus was found to be correlated to worse performance on the virtual maze.

The analysis of signal change revealed that the right lingual gyrus and left fusiform gyrus were more activated in the route than the slanted perspective condition, and higher in turn in the slanted than in the survey condition. The lingual gyrus is part of the occipito-temporal pathway that is engaged by object discrimination and recognition. This activity in the lingual gyrus may reflect the recognition of landmarks or specific intersections that have been coded in an object-like manner [43]. Route and slanted perspective seem likely to involve landmark or intersection recognition to a greater extent than survey perspective. The fusiform gyrus is also involved in landmark identification, probably through geometrical feature analysis [44]. The profile of activation of middle occipital cortex was relatively complex in our study, but may also reflect important specificities of the different perspective conditions. During the encoding task, left middle occipital cortex was less activated in the route perspective condition than in the slanted perspective condition, and less activated with the slanted perspective than the survey perspective. In contrast, in the shortcut task, the reverse pattern was observed, with survey perspective associated to lower activation of middle occipital cortex than slanted perspective, which in turn induced lower activation than route perspective. The middle occipital cortex is involved in the analysis of landmarks and landscapes, and is crucial for viewpoint-independent representation of landmarks [44]. In navigational tasks, this area is thought to be related to visual imagery and, in particular, to the rehearsal of imaginable information [45]. Thus, Nemmi et al. [46] demonstrated that activation in the middle occipital cortex is related to the processing of the visuo-spatial attributes of perceived landmarks and to the rehearsal of the entire route to detect matches and mismatches. Our results suggest that the differential activations of middle occipital cortex, lingual and fusiform gyrus in the three perspective conditions depending on the task may reflect reliance on spatial relationships between landmarks extracted during the path in the process of inferring possible shortcuts. Interestingly, the slanted and survey perspectives induced similar brain changes in left lingual and fusiform gyri but dissociated brain activity changes in these structures in the right hemisphere.

The activation of the left hippocampus observed in our study also suggests that this structure may play an important and differential role in the three perspective conditions. Indeed, although slanted and survey perspectives did not induce differential activity in left hippocampus during the encoding task, the slanted perspective was associated with greater activation of left hippocampus than the survey perspective during the shortcut task. The route perspective, which induced the lowest level of activation during encoding, induced the highest one during the shortcut-finding task. Activation of the right hippocampus has been strongly associated with the accurate determination of locations and accurate navigation between them [36]. Left hippocampus, on the other hand, is probably involved in non-spatial aspects of navigation. Indeed, human left hippocampus may show a preference for episodic, and specifically autobiographic, event memory [47], [48]. Left hippocampus has been found to be activated during retrieval of autobiographical memories [47], [48] as well as during the retrieval of aspects of personally experienced events in a virtual reality environment [15]. Activation of the left hippocampus has also been noted in neuroimaging studies of navigation [8], [38], [43] but this activity was not found to correlate with any navigation measures, unlike right hippocampal activity. This suggests that the left hippocampus may be preferentially (but not exclusively) involved in non-navigational aspects of episodic memory.

To our knowledge, the present study is the first to investigate the brain structures associated with both encoding and shortcut-finding in virtual reality. We observed that the encoding task was specifically associated with occipital and bilateral parietal activations, while the shortcut task elicited activity in a large neural network encompassing frontal, paralimbic, occipital, parietal, temporal cortices and subcortical areas. Parietal activation during path encoding was previously observed by Shelton and Gabrieli [26], who suggested that parietal areas are associated with mental rotation of objects and attention [49], [50].

Our results clearly demonstrated that slanted and survey perspectives share common brain activity in the left lingual and fusiform gyri and lead to very similar behavioural performance. Nevertheless, we observed that slanted perspective induced intermediate patterns of activation (in between route and survey) in some brain areas, such as the right lingual and fusiform gyri. Slanted perspective was also associated with similar activation to route perspective during encoding in the right middle occipital gyrus. These results suggest that the slanted perspective may be considered as a hybrid perspective. However, among the brain structures predicted to be specifically involved in route or survey perspectives [26]–[27], we only observed activations of the medial temporal lobe and the angular gyrus. In these conditions, common activations to survey and slanted perspectives or to route and slanted perspectives were mainly observed in structures that were not specifically activated in the similar previous studies [26], [27]. These results might be explained by the nature of the task involved in our experiment. Indeed, subjects were informed before the encoding phase that they would have to realize a shortcut after the presentation of the path. This condition of encoding differed from the task used in the similar previous studies [26], [27] in which subjects had to encode the path in order to reproduce it. Furthermore, to the best of our knowledge, the brain areas implicated in navigation during a shortcut finding task were not previously investigated.

The shortcut finding task used in our experiment may have favoured reliance on the survey-like aspects of the slanted perspective. Further study involving a spatial task that advantages the route perspective could provide a clear demonstration of the hybrid nature of the slanted perspective. This would provide clear scientific support for the choice to use this perspective in many navigational aids.

In conclusion, we have shown for the first time that slanted and survey perspectives lead to improved performance in finding a shortcut in a virtual reality environment in comparison to route perspective. They induce relatively similar brain activations. These results reveal that a map-like cognitive representation may be built during a single presentation of an environment from a slanted perspective, as it is from a survey perspective.
